# Understanding the social context of fatal road traffic collisions among young people: a qualitative analysis of narrative text in coroners’ records

**DOI:** 10.1186/1471-2458-14-78

**Published:** 2014-01-24

**Authors:** Paul Pilkington, Emma Bird, Selena Gray, Elizabeth Towner, Sarah Weld, Mary-Ann McKibben

**Affiliations:** 1 Faculty of Health and Life Sciences, Department of Health and Applied Social Sciences, University of the West of England, Bristol BS16 1DD, United Kingdom; 2 NHS Wiltshire, Southgate House, Pans Lane, Devizes, Wiltshire SN10 5EQ, United Kingdom

**Keywords:** Road traffic fatalities, Uoung people, Qualitative, Narrative text, Coroners’ records

## Abstract

**Background:**

Deaths and injuries on the road remain a major cause of premature death among young people across the world. Routinely collected data usually focuses on the mechanism of road traffic collisions and basic demographic data of those involved. This study aimed to supplement these routine sources with a thematic analysis of narrative text contained in coroners’ records, to explore the wider social context in which collisions occur.

**Methods:**

Thematic analysis of narrative text from Coroners’ records, retrieved from thirty-four fatalities among young people (16–24 year olds) occurring as a result of thirty road traffic collisions in a rural county in the south of England over the period 2005–2010.

**Results:**

Six key themes emerged: social driving, driving experience, interest in motor vehicles, driving behaviour, perception of driving ability, and emotional distress. Social driving (defined as a group of related behaviours including: driving as a social event in itself (i.e. without a pre-specified destination); driving to or from a social event; driving with accompanying passengers; driving late at night; driving where alcohol or drugs were a feature of the journey) was identified as a common feature across cases.

**Conclusions:**

Analysis of the wider social context in which road traffic collisions occur in young people can provide important information for understanding why collisions happen and developing targeted interventions to prevent them. It can complement routinely collected data, which often focuses on events immediately preceding a collision. Qualitative analysis of narrative text in coroner’s records may provide a way of providing this type of information. These findings provide additional support for the case for Graduated Driver Licensing programmes to reduce collisions involving young people, and also suggest that road safety interventions need to take a more community development approach, recognising the importance of social context and focusing on social networks of young people.

## Background

Globally, road traffic collisions (RTCs) are the leading cause of death in people aged 15–19, and the second highest cause of death in 20–25 year-olds [1]. In the UK around 300 young people aged 16–29 were killed when driving or riding vehicles, and over 4000 seriously injured during 2011 [2]. RTCs are of particular concern in rural areas in the UK, with the highest proportion of RTC-related fatalities and injuries occurring on rural roads [3]. In general, UK figures present a more favourable picture than many other countries; however, recent UK policy documents suggest that more work can be done to reduce road deaths and injuries [4].

Research suggests that there may be specific risk factors for RTCs which are unique to, or elevated in, young people compared with older adults. These include: limited driving experience; night-time driving; fatigue; particular risks for young men [5]. Other age-specific risk factors may include: personality characteristics; driving ability; demographic factors; perceived environment; driving environment; and developmental factors [6].

To develop effective prevention programmes the factors associated with RTCs must be identified and understood. In the UK, routine road traffic injury data are compiled via STATS19, a national database detailing the nature of a collision, the location, and a record of casualty involvement. These data are collected at the scene of the collision by an attending police officer. Although such quantitative data provide valuable intelligence for the monitoring and prevention of RTCs and related casualties, other data sources (including those facilitating qualitative analysis) may complement existing routine data, thus aiding action on road safety [7-9]. For instance, narrative text in particular can provide more detail on the events surrounding unintentional injury and death [8]. However, analysis of narrative text in the injury field has largely sought to quantify that data [8]. Qualitative methods, including thematic analysis of narrative text, offer opportunities for an in-depth examination of phenomena [10]. A qualitative approach can complement quantitative methods, by taking into account the wider social context of the crash and examining attitudes and experiences of those connected to the event.

### Study aims

In other countries, most notably Australia, the value of coroners’ records for informing public health action is recognised, particularly relating to injury prevention [11,12]. Previous studies in England and Wales have examined coroners’ records for public health purposes, but the focus has most often been on suicide prevention rather than issues such as road traffic fatalities [13]. Coroners’ records contain a range of narrative text that is conducive to qualitative analysis, including witness statements, police reports and court transcripts. Therefore, this study used a thematic analysis of narrative text, contained in coroners’ records of fatal RTCs among young people (aged 16–24) in a rural county in the south west of England, to explore if these might complement exist data sources and help identify further areas for prevention.

## Methods

### Population

A rural county located in South West England was selected as the setting for this research as the region has been identified as having a significantly high road fatality rate and local authorities wanted to conduct more research into this area [3,14]. Between 2005 and 2010 in the county, 35 young people aged 16–24 were killed in an RTC, either as a driver of a motorcar or rider of a motorcycle, or as a passenger alongside a person of that age.

### Sample selection

All RTC-related fatalities among 16–24 year old drivers in the selected area occurring between 2005 and 2010 were eligible for inclusion. Passenger fatalities among 16–24 year olds were also eligible, provided the driver of the vehicle was also aged 16 to 24. The age range was selected in response to evidence that drivers aged 16–24 are greatly overrepresented in road death statistics when compared with other, more experienced drivers [15].

### Data collection

Permission to view and extract data from the Coroners’ records was sought and obtained from the coroner for the area. Eligible cases were obtained during visits to HM Coroner, with data extraction taking place on site. A single coroner covered the whole county. Prior to the beginning of the study a qualitative data extraction tool and framework was developed by the research team, following a review of the literature, and tested during a pilot data collection visit to HM Coroner. The tool consisted of a number of headings based on known risk factors identified by Shope [6] during a comprehensive review and synthesis of quantitative and qualitative research findings relating to RTCs among young drivers. Identified risk factors included: personality characteristics (e.g., level of aggression); developmental factors (e.g., sleep patterns); driving ability (e.g., driving skill); demographic factors; perceived environment (e.g., risk perception); and, driving environment (e.g., weather conditions) [6]. The tool was piloted on seven of the RTCs included in this study. Following the guidelines for inductive qualitative research [16,17], this process allowed the researchers to assess whether the pre-specified headings were applicable to the available data, and to identify any additional areas which may provide in-depth rich descriptions which may aid our understanding of fatal RTCs among young people. Four additional headings were identified and added to the tool during the pilot visit: reason for travel; driving behaviour at the time of the collision; safety features of the vehicle; and, vehicle condition. All data extracted at the pilot visit were reviewed in light of the amended data extraction tool during subsequent data collection. Records for each fatality, including police reports, witness statements and the inquest report pertaining to each RTC were reviewed on site in the Coroner’s office over a four day period. Qualitative data concerning each of the framework headings were extracted verbatim from each record directly into a computerised version of the data extraction tool. Demographic data and basic quantitative descriptive data about the RTC, including vehicle details, travel environment, and collision outcomes were also collected from a combination of STATS19 data and information collected from sources in the coroners’ records where available. Where multiple fatalities were recorded from one collision, data on each fatality were extracted. Descriptive information for each case was anonymised at the Coroner’s office. Qualitative data containing information that had the potential to identify a specific individual were anonymised further following discussion among researchers (PP, EB, SG, ET, SW, MM).

### Data analysis

Information recorded in the data extraction form for all cases were imported into NVivo (QSR International 9) verbatim. Each case was explored using Thematic Analysis (TA); a useful method for “identifying, analysing and reporting patterns within data” [18]. One researcher (EB) read through each case multiple times to aid familiarisation. The coding process was predominantly based upon the headings identified in the pre-defined data extraction tool. The data obtained from each case were independently reviewed by one researcher (EB) and the data included under each pre-defined heading of the extraction tool were analysed separately. Notably, the researcher was careful not to be restricted by these pre-defined concepts, allowing for additional codes to emerge from the data inductively. Following this process, the researcher reflected on the codes and allowed wider themes to emerge, with clear examples of each theme taken from the data to illustrate and support the findings of the analysis. To confirm accuracy and interpretation of the data during the coding process and at theme development, findings were discussed and agreed between two researchers (EB and PP) and also among the project steering group (SG, ET, SW, MM).

### Ethical considerations

The Ethics Committee of the University of the West of England declared that no ethical approval was required for this study. Given the highly sensitive nature of the research, care was taken to remove any identifiable content from the data.

### RATS Guidelines

The authors confirm that this study adheres to the RATS guidelines on qualitative research.

## Results

Sample characteristics are available in Table 1. Six key themes emerged. These were: social driving, driving experience, interest in motor vehicles, risky driving behaviour, perception of driving ability, and emotional distress.

**Table 1:** Sample characteristics (n = 34)

		**n**	**%**
Sex	Male	30	88
	Female	4	12
Vehicle type	Car	31	91
	Motorcycle	3	9
Driving status	Driver	29	85
	Passenger	5	15
Vehicle occupancy	Single occupant	19	63
	Passengers present	11	37
Passenger details	1 passenger present	8	73
	2 passengers present	2	18
	4 passengers present	1	9
Presence of alcohol	Yes	11	32
	No	23	68
Presence of drugs	Yes	5	15
	No	29	85
Time of collision	06:00–17:59	13	38
	18:00–05:59	21	62

### Social driving

A particular theme arising from the thematic analysis was what we have termed “social driving”. This term has been used to encompass a group of related behaviours, and included: driving as a social event in itself (i.e. without a pre-specified destination); driving to or from a social event; driving with accompanying passengers; driving late at night; driving where alcohol or drugs were a feature of the journey).

The majority of young drivers were found to be engaged in social driving behaviour prior to, or at the time of, the collision. Many individuals were reportedly travelling with friends to or from a social event, and some of the young people were driving without a specific purpose or destination. Driving without a specific destination appeared to be a specific social activity, allowing the young people to meet up in their cars and socialise together, often occurring at night:

*“He had a hectic social life and almost seemed to be out of the house more than in… once he passed his driving test* [Case 30] *would travel around with his friends.”*

Case 30, Driver, Male

“Usually every weekend I’ll get a call about 2 or 3 o’clock in the morning…saying “Can you come get me?”… So I get up and go and pick them up straight away.”

Case 4, Driver, Male

Nine cases of social driving were from cars which contained at least one passenger. In one case, a driver was transporting passengers home from a social event in the early hours of the morning. The passengers were in high spirits, and were reportedly distracting the driver throughout the journey. The police report stated:

“…that they had been messing about in the back of the car, trying to put their hands over his eyes and slapping his face while he was driving.”

Case 4, Driver, Male

### Driving experience

A number of cases involved people with very limited driving experience, with many passing their driving test within the year prior to their collision. In one case, the driver had only passed their test on the previous day.

One police report stated:

“The driver had limited experience as a driver, having passed his test three months prior to this incident…The driver was confronted with another driver, coupled with a situation that he reacted harshly to, which caused him to lose control of his vehicle.”

Case 18, Driver, Male

In contrast, some individuals were relatively experienced drivers given their age. In six cases, driving experience had been obtained through choice of career as a vehicle driver. One parent reported:

“He had licences in CBT/ATV/HGV and trailer tractors as well as a car. He had a couple of specialist licences, such as forklift and cherry picker.”

Case 3, Driver, Male

Some drivers had limited experience with their vehicle. Over one quarter of cases had been driving the vehicle in which they had their collision for a short time only (weeks or months). In one case, the vehicle had been purchased on the day before their collision. In another case, the driver had recently bought a new vehicle with a large engine size. The driver’s friend commented:

*“Approximately 6 to 8 weeks ago* [he] *purchased a 2.2 litre… he only passed his driving test about 12 months ago.”*

Case 12, Driver, Male

### Interest in motor vehicles

Many young people had a particular interest in motor vehicles. There were several examples when an individual reportedly spent time caring for their vehicle, making sure it was kept in a good quality condition, both aesthetically and mechanically. There were also reports of vehicle modification. In one case, a car had been modified from a 1400 cc engine to a 1600 cc engine. In one statement, a colleague of the deceased reported:

*“He always talked about his car and how he loved to drive it*”

Case 12, Driver, Male

In another example, a Mother stated:

*“It has surprised me that* [Case 15] *has died as a result of a car accident as she would not wish to damage her car in any way. Her car was her pride and joy. She was that particular she would even pick parking spaces away from other vehicles so that it wouldn’t get marked or damaged.”*

Case 15, Driver, Female

### Risky driving behaviour

Dangerous driving behaviour was identified in the majority of cases. This included: driving at speed, tailgating, racing friends and undertaking hazardous overtaking manoeuvres. Notably, excessive speed was referred to in nearly all cases. In one statement, a witness commented:

“My first impression was that it was travelling far too fast to negotiate the bend safely....I could see his hands turning the steering wheel to his right in a large movement, his whole body movement and body language gave me the impression of panic.”

Case 28, Driver, Male

In three cases, it was noted that male driving style may be affected by the presence of other males in the car, causing them to behave differently to normal. In one case, a female friend commented:

*“He always drove safely with me in the car.* [Case 18] *had mentioned that he drove faster when he had the lads in the car. I had not experienced him driving excessively fast myself.”*

Case 18, Driver, Male

### Perception of driving ability

There was a sense of overconfidence and an inflated view of driving skills and ability among many cases. One case concerned two cars that were involved in a race. On interviewing the male driver of the second car, who was unharmed, the police reported:

*“… He agreed that he had been driving 2 to 3 car lengths behind* [Case 15] *at approximately 80 mph. He did not consider this to be an unsafe following distance.”*

Case 15, Driver, Female

In another example, a rear-seat passenger stated:

*“I also saw at least one large arrow shape, indicated to our left. I knew this to mean that we should stay on our own side of the road.* [Front seat passenger] *was shouting “*[Case25]*, what are you doing you are not going to make that” or similar. I became aware that we were now on the offside of the road…As this was happening I heard* [Front seat passenger] *shout a second time. This sounded much more urgent than before as he said, “we’re not going to make that”.”*

Case 25, Driver, Male

The perception of driving ability was linked to a lack of adherence to safety regulations. Although the majority of young people observed safety regulations, the decision not to wear a seatbelt or helmet suggests that such drivers were confident in their ability to drive without incident. In one case, despite being involved in a collision in the week leading up to his death, the driver chose not to wear a seatbelt, as described by a friend:

*“I can say that* [Case 13] *was not in the habit of wearing a seatbelt as he found it too restrictive. I had asked him if he was wearing one when he hit the van* [referring to prior collision]*. He said he had not but had been able to brace himself on that occasion against the steering wheel.”*

Case 13, Driver, Male

There were many instances where statements contained others’ perceptions of the driver’s driving ability. People’s perceptions of the deceased’s driving ability were shown to differ; in some statements, people were highly complementary, while other statements highlighted a less favourable view, and in some cases were visibly critical of the deceased’s driving ability. The majority of statements presented a positive perception of the driving ability of the deceased. This is interesting, given the knowledge that many collisions were associated with risky or dangerous driving behaviour. The partner of one individual reported:

*“*[Case 24] *was an excellent driver especially considering that he was still young and didn’t have a lot of experience. He never drove fast with me or* [their baby] *in the car and certainly wouldn’t do if the roads were potentially risky. He constantly talked about other accidents he’d seen to and from work, which always reassured me that he’d drive safely.”*

Case 24, Driver, Male

However, other statements were not so positive. One father reported of his son:

“He was in my eyes a typical young driver. He had a few bumps and things. I would say he was a confident driver but at times over confident. He sometimes drove and I would say stop, drop me off. I think his driving just needed maturity.”

Case 21, Driver, Male

### Emotional distress

In our study emotional distress immediately prior to the RTC was identified in over one quarter of the cases. This mainly referred to family or personal relationship problems, or financial difficulties, issues which may have distracted the driver from their driving role. In one case, a driver was struggling to cope with relationship and financial demands. As one police report stated:

*“It is highly likely on the evidence available that* [Case 24] *was in a hurry to get home…it is possible there were other things on his mind, for example the issue of the rent on his flat.”*

Case 24, Driver, Male

A further statement, provided by the mother of the deceased reported:

*“*[Case 24’s]…*relationship with…* [His girlfriend] *was very rocky throughout the time they were together. On the Sunday before the accident he was at our house with all of his stuff saying that he’d had enough of his relationship with…* [his girlfriend] *and wanted to return home.”*

Case 24, Driver, Male

Relationship difficulties were identified from a further 6 cases. In one passenger fatality a police report stated:

*“*[The driver] *is married and* [Case 9] *has recently separated from her long term boyfriend.* [The driver] *and* [Case 9] *have been involved in a relationship for some weeks preceding the collision.* [The driver] *had informed his wife of his affair on* [the day prior to the collision] *when his wife asked him to leave their marital home.”*

Case 9, Passenger, Female

## Discussion

### Main findings

Thematic analysis identified six themes: social driving, driving experience, interest in motor vehicles, risky driving behaviour, perception of driving ability, and emotional distress. Notably, multiple themes were identified from each case, indicating that there may be numerous factors which influence the causes and characteristics of RTCs among young people. The findings of the present study were consistent with previous research identifying risk factors concerning RTCs involving young people [1,6,19-22].

The high prevalence of social driving among young people is likely to relate to the rural setting where the study took place, and may not be replicated in urban areas. Unlike urban dwellers, people who live in a rural area often have limited access to public transport and therefore need a car for travel. For young people in particular, transport is required for school, work and maintaining a social life [23]. In this study, social driving behaviours referred to: driving as a social event in itself (i.e. without a pre-specified destination); driving to or from a social event; driving with accompanying passengers; driving late at night; or driving where alcohol or drugs were present. The numerous attributes associated with social driving behaviour highlight the difficulties in understanding the impact of different factors leading up to a fatal RTC. The association between driving and the social environment has received some research attention in recent years; for example, the influence of passengers upon driving ability [6]. However, there has been less consideration of “social driving” as a driving ‘culture’ among young adults [24], as observed in the present study.

Risky driving behaviour was another regular feature of RTCs among young people in the study area, and is a finding that is consistent with previous research [20,25,26]. Risky driving behaviour is often associated with driving inexperience; young people have been shown to underestimate risk and overestimate their driving ability [27,28]. Although the acquisition of driving knowledge and skills are important if competent driving ability is to be achieved, it is also essential to obtain driving experience through practice [6].

Emotional distress at the time, or leading up to, the collision was identified from numerous cases. Previous studies investigating the causes and characteristics of RTCs have indicated that psychological factors may influence driving behaviour in young people [6,29-31]. The results from previous research imply that interventions could be targeted towards specific ‘at-risk’ groups, or possibly that emotional distress could be included in general road safety awareness campaigns; for example, warning people about the dangers of driving when emotionally distracted. It was notable that the mental distress identified in the current study was often triggered by a recent traumatic event.

### Implications of the findings

The finding that groups of young people engage in social driving behaviour, and the existence of large social networks of young drivers in and around the rural market towns, lends support to the development of interventions that target these groups using a multi-strategy approach in their specific social context. There is evidence that such community based interventions have the potential to reduce child and adolescent unintentional RTC injuries [32]. They can include interventions such as education, legislation, social and environmental interventions [33] in an attempt to change community norms and behaviour. Given the limited evidence of effectiveness of educational training alone [24], and that education-based training interventions have also been criticised for failing to address the social and lifestyle factors that are associated with risky driving behaviour among young people [34], there would seem to be a need to develop novel community-based interventions targeted at a specific group of young drivers.

The findings of this study also lend support to prevention programmes such as Graduated Driver Licensing (GDL), which have been effective in reducing young driver collision rates in parts of the USA, Canada, Australia and New Zealand [1,35,36]. GDL schemes involve restrictions for newly qualified drivers which may include: limitations on accompanying passengers, restrictions on night-time driving, and lower thresholds for blood alcohol concentration. In the UK it is predicted that such a programme (night-time restrictions between 9 pm-6 am, and no 15–24 year old passengers) could save more than 114 lives and result in 872 fewer serious injuries each year among younger drivers [37]. In the current study, if night-time restrictions had been imposed at the time of these collisions, it is estimated that 27% of causalities may have been prevented. The ability of GDL to disrupt the social networks identified in this research is also significant. However, given the rural nature of the study area, legislation restricting night-time and passenger journeys may be difficult to enforce and also result in young people becoming socially isolated and unable to participate in school or work activities [38]. An ideal GDL approach would minimise such costs, while maximising the potentially significant benefits in terms of preventing deaths and injuries on the road.

Parental supervision of young drivers could provide an alternative approach to restricting driving through legislation as parents may have the ability to moderate their child’s driving behaviour and may contribute to decisions or funding about vehicle purchase [39]. However, as observed in the present study, parents often perceived their child to be a competent, responsible driver, despite many of the collisions being associated with risky driving behaviour. Further, while parents appear to be aware of the risks associated with new drivers, they are also keen to encourage independence in their children and reduce the need to transport them to various events [39]. A small number of interventions have investigated the impact of parental supervision, resulting in somewhat mixed overall findings [40,41]. Further exploration of interventions involving parental supervision would therefore be desirable, but at present they do not appear to be an adequate substitute for GDL.

### Strengths and limitations

To our knowledge, this is the first study to undertake a detailed qualitative thematic analysis of narrative text contained in coroners’ records of fatal RTCs among young people. The records provided a comprehensive report on each collision, and the majority of case files contained collision information that went beyond consideration of the physical crash. In some post-collision interviews with family or friends of the deceased, the wider social context of the collision was explored with inclusion of, for example, details of family circumstances. The records therefore complemented routinely collected STATS19 data, which focus primarily on physical crash conditions and acute actions immediately before the collision.

Although the number of cases was relatively small, we did feel that we achieved saturation for identification of themes, and the qualitative nature of the study allowed for a thorough, in-depth analysis of cases in a way that is not routine in road fatality analysis. Comparison with existing theoretical models and literature on risk factors for young people allowed us to locate our findings within a wider UK and international context, adding to the existing evidence base.

The study underlines the important role that in-depth data sources such as coroners’ records can play in informing prevention efforts. Countries should facilitate easier access to such data, as has happened in Australia with the development of the National Coroners Information System [42]. The current system of multiple autonomous coroners leads to under-utilisation of coroners’ data in the UK and elsewhere, representing a missed opportunity for public health [43].

## Conclusions

This study used qualitative thematic analysis of narrative text in coroners’ records to examine the social context of fatal road traffic collisions in young people. It found themes consistent with other frameworks [6], but used the richness of the qualitative data to examine in greater depth the previously identified risk factors. These in-depth findings provide additional support for the case for Graduated Driver Licensing programmes to reduce collisions involving young people, and also suggest that road safety interventions need to take a more community development approach, recognising the importance of social context and focusing on social networks of young people. Further research in the UK and other countries could replicate this approach, to build on the work presented here and the pre-existing frameworks.

## Competing interests

The authors declare that they have no competing interests.

## Authors’ contributions

The study was conceived by PP. PP and EB were responsible for data collection. PP and EB analysed the data and discussed the findings with SG, ET, SW, and MM. PP drafted the first version of the manuscript. All authors provided critical edits and revisions to the manuscript, and reviewed and approved the final version.

## Pre-publication history

The pre-publication history for this paper can be accessed here:

http://www.biomedcentral.com/1471-2458/14/78/prepub
